# Extinction of cue-evoked drug-seeking relies on degrading hierarchical instrumental expectancies

**DOI:** 10.1016/j.brat.2014.06.001

**Published:** 2014-08

**Authors:** Lee Hogarth, Chris Retzler, Marcus R. Munafò, Dominic M.D. Tran, Joseph R. Troisi, Abigail K. Rose, Andrew Jones, Matt Field

**Affiliations:** a School of Psychology, University of Exeter, Washington Singer Building, Perry Road, Exeter EX4 4QG, UK; b School of Psychology, University of New South Wales, Sydney, NSW 2052, Australia; c Department of Behavioural and Social Sciences, University of Huddersfield, Huddersfield, UK; d MRC Integrative Epidemiology Unit and School of Experimental Psychology, 12a Priory Road, University of Bristol, Bristol BS8 1TU, UK; e Department of Psychology, Saint Anselm College, Manchester, NH 03102, USA; f School of Psychology, University of Liverpool, Liverpool, L69 7ZA, UK; g UK Centre for Tobacco and Alcohol Studies, UK

**Keywords:** Extinction, Learning, Transfer, Dependence, Relapse

## Abstract

There has long been need for a behavioural intervention that attenuates cue-evoked drug-seeking, but the optimal method remains obscure. To address this, we report three approaches to extinguish cue-evoked drug-seeking measured in a Pavlovian to instrumental transfer design, in non-treatment seeking adult smokers and alcohol drinkers. The results showed that the ability of a drug stimulus to transfer control over a separately trained drug-seeking response was not affected by the stimulus undergoing Pavlovian extinction training in experiment 1, but was abolished by the stimulus undergoing discriminative extinction training in experiment 2, and was abolished by explicit verbal instructions stating that the stimulus did not signal a more effective response-drug contingency in experiment 3. These data suggest that cue-evoked drug-seeking is mediated by a propositional hierarchical instrumental expectancy that the drug-seeking response is more likely to be rewarded in that stimulus. Methods which degraded this hierarchical expectancy were effective in the laboratory, and so may have therapeutic potential.

## Highlights

• Reveals why drug cue extinction therapy has been clinically ineffective.
• Provides laboratory validation of a more effective extinction method.
• Clarifies the associative mechanisms underpinning drug cue reactivity & extinction.
• Corroborates human and animal data.
• Discusses the therapeutic potential of the new extinction method.

## General introduction

Drug-related stimuli provoke craving (Sayette & Tiffany, 2013), drug-seeking (Hogarth & Chase, 2011) and drug-taking behaviour (Hogarth, Dickinson, & Duka, 2010), and contribute to the maintenance and relapse to drug use in the natural environment (Shiffman, 2009). Based on principles of associative learning (e.g. Hogarth, Balleine, Corbit, & Killcross, 2013), therapeutic interventions have sought to extinguish either the Pavlovian contingency between drug stimuli and drug outcome (S-O) through presentation of the S in the absence of the O (Pavlovian extinction), or extinguish the instrumental contingency between the drug-taking response and the drug outcome (R-O) by the performance of ‘mock’ drug-taking in the absence of the O (instrumental extinction). Although such extinction procedures reduce cue-evoked craving in the laboratory, they produce no long term effects on abstinence in the field (Collins & Brandon, 2002; Conklin & Tiffany, 2002; Price et al., 2010; Thewissen, Snijders, Havermans, van den Hout, & Jansen, 2006; Xue et al., 2012).

One explanation for the clinical failure of extinction training is that nonreinforcement of the binary S-O or R-O contingencies does little to modify the discriminative or hierarchical function of drug stimuli in signalling the current strength of the response-drug contingency, S:R-O (Bradfield & Balleine, 2013; Colwill, 1994; Rescorla, 1991, 1992b). According to the hierarchical account, drug stimuli retrieve an expectancy that the drug-seeking response will produce the drug, which primes performance of the response. For example, seeing a pub retrieves an expectation that specific entry and purchasing behaviour will produce a drink for consumption, and this gestalt expectancy of the response-outcome chain retrieved by the stimulus promotes initiation of the entry and purchasing behaviour. If cue-provoked relapse is mediated by such a hierarchical expectancy that in the presence of the stimulus the drug-seeking response will be effective (S:R-O), then learning in Pavlovian extinction that a drug stimulus is not followed by the drug (S-no O) in the absence of a drug-seeking response, or learning in instrumental extinction that a drug-seeking response fails to produce the drug (R-no O) in the absence of drug stimuli, should be expected to have little therapeutic impact on cue-provoked relapse. In short, the hierarchical expectancy controlling relapse cannot be abolished by degrading its component Pavlovian and instrumental parts in isolation. Accordingly, extinction procedures which specifically degrade the hierarchical function of drug cues, such that the cues no longer signal a stronger response-drug contingency, may provide a better therapeutic solution (Conklin & Tiffany, 2002 page 163). The current study tested this proposal (Gass & Chandler, 2013).

The experiments reported here measured cue-evoked drug-seeking in a specific Pavlovian to instrumental transfer (PIT) procedure because this procedure is largely unique in being able to isolate the control exerted by stimuli over instrumental responses controlled by knowledge of specific R-O contingencies (isolated from control by habitual stimulus-response [S-R] contingencies). In the PIT procedure, two stimuli are paired with distinct rewarding outcomes, S1-O1, S2-O2, using either Pavlovian or discriminative instrumental training, and separately, two instrumental responses are trained for the same outcomes, R1-O1, R2-O2. In the transfer test, the stimuli are presented for the first time while the two responses are available, and a specific PIT effect is demonstrated when each stimulus selectively enhances responding for the same outcome (i.e. S1: R1 > R2, S2: R1 < R2). This effect has been found in both humans (Hogarth, Dickinson, Wright, Kouvaraki, & Duka, 2007) and animals (Holmes, Marchand, & Coutureau, 2010) and can be attributed to the hierarchical function of each stimulus activating an expectancy that the R-O pair with the corresponding O has a higher probability of being reinforced in the PIT test, i.e. S:R-O (Bradfield & Balleine, 2013; Colwill, 1994; Rescorla, 1991, 1992b). This S:R-O expectancy may be encoded in associative terms where the S retrieves a gestalt representation of the response sequence followed by the outcome, and/or in propositional terms, where the S retrieves a verbalizable belief that a given response is more likely to produce the outcome (Heyes & Dickinson, 1990). Behavioural economic theory contains a related depiction where stimuli carry information about the probability of a particular response producing an outcome, which is commensurated with outcome value, response effort etc. to produce a relative utility estimates which determines performance of the response (Cisek, 2007).

Studies that have tested the impact of ‘extinction’ procedures (broadly defined) on the PIT effect have produced an erratic pattern of results, which may be explained (at least partially) by these designs having differentially targeted the hierarchical function of stimuli during extinction. Specifically, the PIT effect remains intact despite Pavlovian extinction of the stimuli prior to the transfer test in both animals (Delamater, 1996; Rescorla, 1992a) and humans (Rosas, Paredes-Olay, García-Gutiérrez, Espinosa, & Abad, 2010). Also, Pavlovian counterconditioning of the stimuli has no impact on the PIT effect in animals (Delamater, 1996; Rescorla, 1992a; Troisi, 2006), but oddly, does attenuate the effect in humans (Rosas et al., 2010). By contrast, contingency degradation produced by non-contingent presentation of the outcome during the inter-trial-interval of Pavlovian training does attenuate the PIT effect in animals (Delamater, 1995). However, the most direct finding for the current study is that *discriminative extinction training* in humans abolished the PIT effect (Gámez & Rosas, 2005). In this important procedure, a stimulus was trained to signal one R-O contingency and then in extinction was switched to signal that the R would no longer produce its' outcome. This completely abolished the ability of the S to prime performance of another response trained with the same outcome when they were presented together for the first time in the PIT test.

The current manuscript sought to extend this work by testing whether Pavlovian extinction (experiment 1) is less effective at abolishing the PIT effect produced by drug cues than discriminative extinction training (experiment 2). Furthermore, experiment 3 tested whether instructions degrading propositional beliefs about the hierarchical signalling function of the drug stimulus would similarly abolish the PIT effect. Collectively, these studies would suggest that drug stimuli control drug-seeking by evoking a propositional belief that the drug-seeking response has a greater likelihood of being reinforced, and that cue-exposure therapies which degrade this expectancy might offer a more effective therapeutic solution.

## Experiment 1 – Pavlovian extinction (S-no O)

### Method

Experiment 1 tested whether a Pavlovian extinction procedure (Hogarth & Chase, 2012) would abolish the PIT effect produced by drug cues (Hogarth et al., 2007). We used convenience sampling to recruit 43 smokers of any level of nicotine dependence or satiety, and did not constrain these variables because they have been found to be unrelated to the PIT effect (Hogarth, 2012; Hogarth & Chase, 2011, 2012). Of 43 smokers tested, data from 33 were analysed following exclusion of 10 participants who reported inaccurate knowledge of the instrumental contingencies (*n* = 4) or Pavlovian (*n* = 6) contingencies. The final sample for analysis comprised 52% males, had a mean age of 20 (sd = 2.46), and currently smoked 5.8 (1.65) days per week, 6.4 (3.71) cigarettes per day, had been smoking for 3.9 (2.76) years, and were cigarette deprived an average of 17.3 (23.5) hours. Participants provided informed consent and were paid £5 or received course credits for participation, and the study was approved by the school of psychology ethics committee.

#### Concurrent training

In concurrent training (see Table 1), participants learned to press the D or H key to earn tobacco or chocolate respectively. Each trial presented the prompt ‘Choose a key’, upon which participants pressed the D or H key. One key produced the outcome ‘You win ¼ of a cigarette’, whereas the other produced ‘You win ¼ of a chocolate bar’, each with a 50% probability. ‘You win nothing’ was presented on remaining trials, to facilitate switching between the two responses over 48 trials (response-outcome assignment was counterbalanced between-subjects). At the end, a cumulative total screen instructed participants to cache earned cigarettes and chocolates from two containers holding 10 Marlboro Lights cigarettes and 10 Cadbury Dairy Milk treat-size bars, respectively, into empty containers labelled as the subjects' own. Immediately afterwards participants reported which response they believed produced which outcome (four participants were excluded for inaccurate instrumental knowledge).

#### Pavlovian acquisition

Pavlovian acquisition then established two CS^+^s which were predictive of tobacco and two CS^+^s which were predictive of chocolate. In each trial, one of four letter stimuli (A, B, C or D) was presented and participants were asked ‘What do you think you will win? 1 = Don't know, 2 = Nothing, 3 = Cigarettes, 4 = Chocolate’. Feedback for participants' prediction was either ‘correct’ plus a high pitched beep (22,050 Hz, 500 ms) or ‘incorrect’ plus a low pitched beep (44,100 Hz, 500 ms) for 1 s. The trial outcome then followed; either ‘You win ¼ of a cigarette’ or ‘You win ¼ of chocolate’ presented for 2 s. Over 48 trials, each letter stimulus was presented 12 times in random order (stimulus-outcome contingencies were counterbalanced between-subjects). Knowledge of these Pavlovian contingencies was demonstrated by making a correct prediction in the last two presentations of each stimulus (where the chance level is 1/32; Six participants were excluded for inaccurate Pavlovian knowledge).

#### Pavlovian extinction

**Fig. 1:**
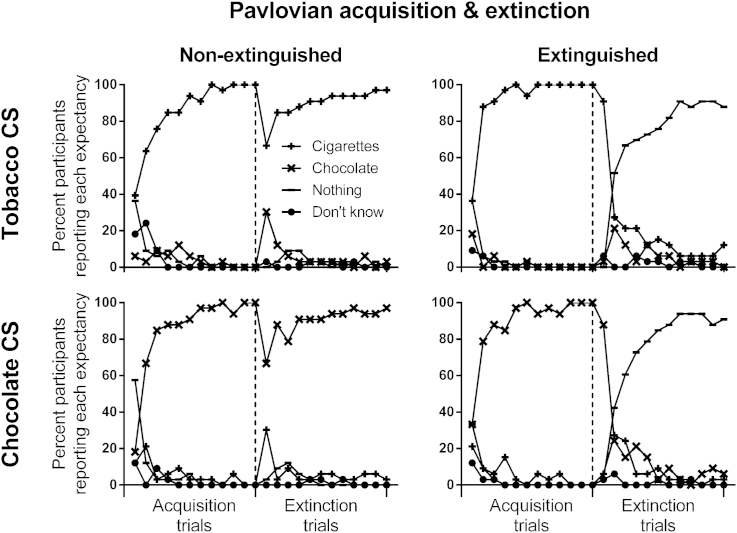
Percent participants predicting the possible outcomes (‘Cigarettes’, ‘Chocolate’, ‘Nothing’, ‘Don't know’) in each of four stimuli. In Pavlovian acquisition, two tobacco CSs and two chocolate CSs predicted their respective outcomes, whereas in Pavlovian extinction, one of each CS type was switched to predict nothing. Participants' predictions closely reflected these scheduled contingencies.

The Pavlovian extinction stage involved scheduling one tobacco CS^+^ and one chocolate CS^+^ as predictors of non-reward (CS^−^s). Training proceeded without interruption from Pavlovian acquisition for another 12 trials of each of the four stimuli, except that one tobacco CS^+^ and one chocolate CS^+^ was now followed by the outcome ‘You win nothing’ (notated as CS^+/−^), whereas other two CS^+^s predicted the same outcomes as before (notated as CS^+/+^). Expectancy reports should reflect this change in the predictive status of CS^−^'s (see Fig. 1).

#### Transfer test

**Fig. 2:**
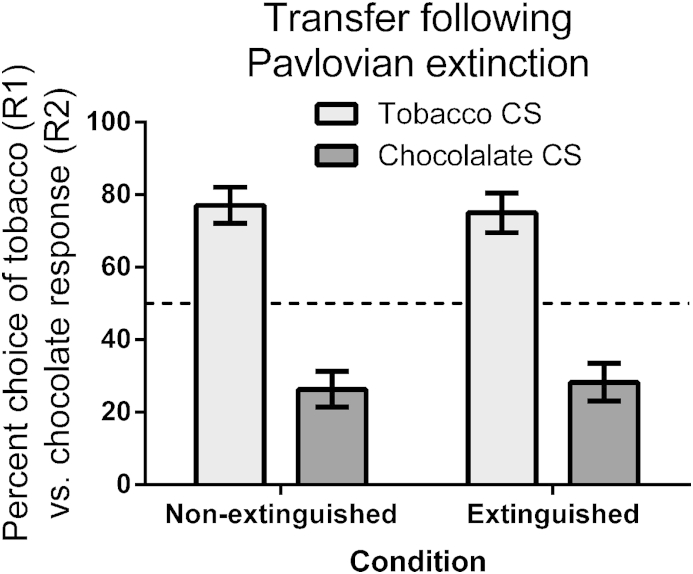
Mean percent choice between a tobacco (R1) vs. chocolate response (R2) (±sem) in the four CSs from Pavlovian training, in the PIT test. The horizontal dashed line at 50% represents indifference between the two responses.

Finally, in the transfer test, participants were told that they could earn cigarettes and chocolate by pressing the D and H keys as earlier in the study, but they would only be told how many of each reward they had earned at the end of the task. This nominal extinction condition was employed to ensure that the specific PIT effect was due to the CS retrieving the relevant R-O contingency, and not by S-R/reinforcement learning within the test phase (as is standard for the PIT procedure in humans and animals; (Hogarth et al., 2013)). In each trial of the PIT test, a CS (A, B, C, D) was presented for 1-s before the prompt ‘Choose a key’ appeared. There were 16 trials of each stimulus (64 trials in total) randomly selected. The dependent measure was the percentage of tobacco vs. chocolate responses in the presence of each stimulus. The question at stake was whether Pavlovian extinction would attenuate the ability of stimuli to promote selective responding for the corresponding outcome (Fig. 2).

### Results

Across Pavlovian acquisition and extinction training, participants' predictive knowledge of the four stimuli accurately traced the scheduled contingencies, as shown in Fig. 1. In acquisition, all participants rapidly learned to predict the correct outcome (recall that participants were only included in this analysis if they demonstrated accurate Pavlovian knowledge in the last two acquisition trials of each stimulus). Most importantly, in extinction, practically all participants (≈90%) learned to predict ‘Nothing’ when presented with the two CS^−^s. It is surprising given this knowledge of the nonreinforcement following the CS^−^s, that there was no reduction in the ability of these stimuli to produce a PIT effect compared to the CS^+^s, as shown in Fig. 2. ANOVA on the PIT data yielded a main effect of stimulus, *F*(1, 32) = 27.72, *p* < .001, indicating that the tobacco CS primed the tobacco response, and the chocolate CS primed the chocolate response. However, there was no main effect of extinction, or interaction between CS and extinction, *F*s < 1, indicating that extinction had no impact on the ability of CS to prime responding for the corresponding outcome.

### Discussion

Experiment 1 found that Pavlovian extinction did not abolish the PIT effect produced by drug cues. This finding is consistent with previous studies in which Pavlovian extinction has similarly failed to abolish the PIT effect produced by natural reward paired cues in animals (Delamater, 1996; Rescorla, 1992a) and humans (Rosas et al., 2010). These studies support Conklin and Tiffany's (2002) claim that degrading Pavlovian contingencies alone is not sufficient to abolish cue-evoked drug-seeking. The question is what extinction method will abolish cue-evoked drug-seeking.

## Experiment 2 – discriminative extinction (S:R-no O)

### Introduction

Experiment 2 was based on a study by Gámez and Rosas (2005, experiment 2) which abolished the PIT effect with non-drug cues using a discriminative extinction procedure. Recall that Gámez & Rosas (2005) trained an arbitrary stimulus to signal that one R-O contingency was in force S:R1-O1, and then in discriminative extinction switched this stimulus to signal that the R would no longer produce its' outcome S:R1-no O1. Another second response was then trained with the same outcome (R3-O1), before the extinguished S was tested for the ability to prime selection of that new response in a PIT test, S:R3-. The results showed that discriminative extinction abolished the PIT effect produced by this stimulus, suggesting that the acquired S:R1-no O1 extinction rule was inferred to operate in relation to the new response, S:R3-no O1 (Table 2).

Experiment 2 used a related design in which a pictorial beer stimulus underwent discriminative extinction training in which it signalled that a previously established beer response (R1) would be ineffective S:R1-no O1. A new beer response (R3) was then trained, before the ability of the extinguished beer stimulus to prime the new beer response was assessed in a PIT test, S:R3-, compared to a group that had not received discriminative extinction training. It is expected that discriminate extinction training will abolish the PIT effect produced by the beer stimulus, suggesting generalisation of the S:R1-no O1 rule to the new beer response, S:R3-no O1. This finding would provide insight into a more effective therapeutic strategy than is offered by Pavlovian extinction.

### Method

The sample of forty social drinkers (20 per group: extinguished vs. non-extinguished) comprised 33% males with a mean age of 19 (sd = 1.92) and an Alcohol Use Disorder Identification Test (AUDIT) score of 7.8 (5.42), which is just below the hazardous categorization score of 8 (Babor, Higgins-Biddle, Saunders, & Monteiro, 2001). Convenience sampling was again employed with the only restriction being that participants reported some level of drinking (i.e. were not teetotal), as alcohol dependence has been shown to be unrelated to the alcohol PIT effect (Martinovic et al., 2014). There were no significant differences between the two groups with respect to these three characteristics. Participants provided informed consent and were paid $10 or received course credits for participation, and the study was approved by the school of psychology ethics committee.

#### Concurrent training stage 1

In concurrent training stage 1 (see Table 2), participants learned to press the up and down arrow keys to earn beer or chocolate respectively (2 bottles of chilled Corona Extra beer 330 ml and 2 bars of Cadbury Dairy Milk 230 g were present on the table). Each trial presented the prompt ‘UP or DOWN?’, upon which participants pressed the up or down arrow key. One key produced the outcome ‘One beer point’ accompanied by a picture of a single Corona Extra beer bottle 330 ml, whereas pressing the other key produced the outcome ‘One chocolate point’ accompanied by a picture of a Cadbury Dairy Milk chocolate bar 230 g, each with a 100% probability. There were 20 trials and the response-outcome assignment was counterbalanced between-subjects.

#### Discriminative extinction training

**Fig. 3:**
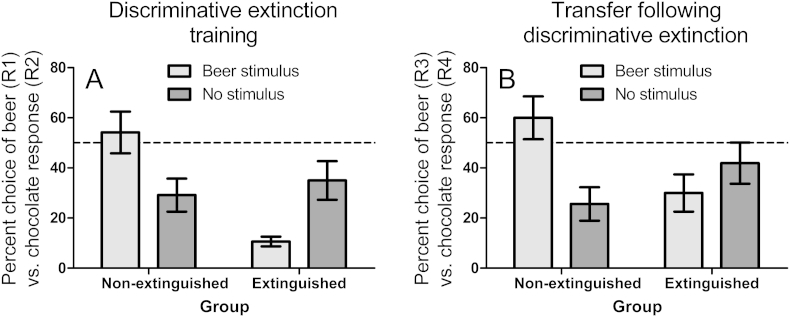
(A) Mean percent choice between a beer response (R1) vs. chocolate response (R2) (±sem) during discriminative extinction training. For the non-extinguished group, both responses were reinforced in both the beer stimulus and no stimulus conditions, yet the beer stimulus primed the beer response. For the extinguished group, the beer stimulus selectively signalled that the beer response would not be reinforced, and came to suppress the beer response. (B) Percent choice between a new beer response (R3) vs. chocolate response (R4) (±sem) in a PIT test in the presence of the beer stimulus and no stimulus established in training. The suppressive effect of the beer stimulus on beer responding acquired by the extinguished group in training with R1, generalized to the new beer response R3 in the transfer test.

In the discriminative extinction training stage, half of participants underwent extinction while the other half did not (Table 2). The purpose of discriminative extinction training was to establish a beer stimulus (two glass jugs of beer being clashed together) as a signal that the beer response (R1) from concurrent training session 1 would now *not* produce beer points (points were used rather than quarters of reinforcers to align the design with the seminal study of Gámez and Rosas (2005) which used game points). Concurrent training proceeded as before for 48 trials except in a randomly selected half of trials, the beer stimulus (S1) was presented alongside the prompt ‘UP or DOWN?’, and pressing the beer key unexpectedly produced the outcome ‘Nothing’ alongside a large red cross stimulus to highlight non-reward. By contrast, pressing the chocolate response (R2) in the beer stimulus continued to produce the outcome ‘One chocolate point’ accompanied by a picture of a Cadbury Dairy Milk chocolate bar 230 g, as before. In the randomly interleaved ‘no stimulus’ trials (S2), both R1 and R2 continued to produce their outcomes as in concurrent training stage 1. Thus, the beer stimulus was scheduled as a unique signal that the beer response would not produce beer. By contrast, in the non-extinguished group, both R1 and R2 continued to be reinforced as before, in both the beer stimulus and no stimulus trials. The dependent measure was the percentage choice of the beer (R1) versus the chocolate (R2) response. It was expected that the extinguished group would learn to withhold the beer response in the beer stimulus, whereas the non-extinguished group would preferentially perform the beer response in the beer stimulus (Fig. 3A).

#### Concurrent training stage 2

The purpose of the concurrent training stage 2 was to establish two new responses, R3 and R4, for beer and chocolate, respectively, using an identical procedure to concurrent training stage 1 except with the A and D key, rather than the up and down arrow key. The R-O assignment was counterbalanced between-subjects with respect to the R-O assignment in concurrent training stage 1. All participants possessed accurate knowledge of the instrumental contingencies involving the A and D key when tested at the end of this stage.

#### Transfer test

Finally, the transfer test assessed whether the capacity of the beer vs. no stimulus to prime choice of the new beer response R3 over the chocolate response R4 (the A or D key) would be reduced as a result of previous discriminative extinction training (beer S:R1-no beer). Participants were told at the outset of the transfer test that they could continue to earn beer and chocolate by pressing the A and D key as before, but they would only be told how many of each reward they had earned at the end (a nominal extinction test procedure identical to experiment 1). In each trial, the prompt ‘A or D?’ was accompanied by either the beer stimulus or no stimulus, randomly selected over 48 trials. The dependent measure was the percentage choice of the beer (R3) vs. chocolate response (R4). It was expected that the beer stimulus would show reduced capacity to elicit the beer response (R3) in the extinguished group (Fig. 3B) demonstrating generalisation of a discriminative extinction rule, S:R1-no O1, to a new response, S:R3-no O1.

### Results

#### Discriminative extinction training

Fig. 3A shows that in the discriminative extinction training stage, the extinguished group learned to suppress beer response in the beer stimulus compared to the non-extinguished group, for whom the beer stimulus primed beer choice. Confirming this description, ANOVA on the data in Fig. 3A produced a significant interaction between stimulus and extinction group, *F*(1,38) = 31.67, *p* < .001. The main effect of stimulus was significant in both the non-extinguished, *F*(1,19) = 19.82, *p* < .001, and extinguished group, *F*(1,19) = 13.07, *p* = .002, and the main effect of extinction group was significant for the beer stimulus, *F*(1,38) = 26.08, *p* < .001, but not for no stimulus, *F* < 1.

#### Transfer test

Fig. 3B shows that the beer stimulus transferred its' suppressive effect acquired in discriminative extinction training to the new beer response (R3) in the transfer stage. ANOVA on Fig. 3B yielded a significant interaction between stimulus and group, *F*(1,38) = 16.07, *p* < .001. The main effect of stimulus was significant for the non-extinguished group demonstrating the standard PIT effect, *F*(1,19) = 17.40, *p* = .001, but was not significant for the extinguished group, *F*(1,19) = 2.16, *p* = .16, demonstrating abolition of the PIT effect. The main effect of extinction group was significant for the beer stimulus, *F*(1,38) = 6.98, *p* = .01, but not for no stimulus, *F*(1,38) = 2.35, *p* = .13.

### Discussion

Experiment 2 found that discriminative extinction training abolished the PIT effect produced by drug cues, as has been found previously with natural reward cues in humans (Gámez & Rosas, 2005). These data suggest that knowledge of one discriminative/hierarchical extinction rule, S:R1-no O1, may be inferred to prevail when another, separately acquired response for the same outcome (R3-O1) is interposed, such that participants infer S:R3-no O1 without having directly experienced extinction of this hierarchical relation. By contrast, Experiment 1 and corroboratory studies (Delamater, 1996; Rescorla, 1992a; Rosas et al., 2010), found evidence that knowledge of a Pavlovian extinction rule, S-no O1, leaves intact the inference of a hierarchical relation, S:R1-O1, when a separately acquired response for the same outcome is interposed. The implication is that focusing on degrading the hierarchical function of stimuli would provide a more effective behaviour therapy than Pavlovian extinction.

## Experiment 3 – hierarchical instructions

### Introduction

A propositional account was offered to explain the impact of discriminative extinction training on PIT. On this view, discriminative extinction training established a veridical belief that in the beer stimulus, the beer response would not be rewarded, S:R1-no O1, which generalized to the new beer response, S:R3-no O1, tested in the PIT phase. To test this propositional account, experiment 3 created a hierarchical extinction rule through verbal instructions. Specifically, prior to the PIT test, half of participants were told that the stimuli did not signal which response was more likely to be rewarded, and afterwards, participants were questioned about their hierarchical beliefs during the PIT test. It was expected that instructions would abolish both hierarchical beliefs and the PIT effect. This correspondence would suggest that drug cues prime drug-seeking by retrieving an expectation that response-drug contingency will be rewarded, and that abolishing this expectation can effectively abolish cue-evoked drug-seeking (Table 3).

### Method

Of thirty social drinkers tested, one was excluded for having an outlying transfer effect (greater than 1.5 times the interquartile range below the mean, which no participant in previous studies had shown) providing 29 participants for analysis (14 instructed, 15 non-instructed). The sample for analysis comprised 46% males, had a mean age of 23 (sd = 3.04) and an AUDIT score of 11.3 (5.3) which is above the hazardous threshold of 8 but below the alcohol dependence threshold of 13 and 15 for females and males, respectively. There were no significant differences between the two groups with respect to these characteristics. Participants provided informed consent and were paid £5 or received course credits for participation, and the study was approved by the school of psychology ethics committee.

#### Concurrent training

In concurrent training (see Table 3), participants learned to press the left and right arrow keys to earn beer or chocolate respectively (3 bottles of Becks beer 330 ml and 3 bars of Cadbury Dairy Milk 49 g were present on the table. Three bottles of beer were used in Experiment 3, rather than two, to try and increase the relative value of beer to bring overall beer choice closer to 50%). Each trial presented the prompt ‘← or →’, upon which participants pressed the left or right arrow key. One key produced the outcome ‘One beer point’, whereas pressing the other key produced the outcome ‘One chocolate point’, each with a 50% probability. There were 24 trials and the response-outcome assignment was counterbalanced between-subjects. All participants possessed accurate knowledge of the instrumental contingencies when tested at the end of this stage.

#### Instructions and transfer test

In the transfer test, the instructed group was told: ‘In this part of the task, you can earn beer and chocolate by pressing the left or right arrow key in the same way as before. You will only be told how many of each reward you have earned at the end of the experiment. Also, sometimes a picture of beer or chocolate will be presented before you choose the left or right arrow key. PICTURES DO NOT INDICATE WHICH ARROW KEY IS MORE LIKELY TO BE REWARDED! Press any key to begin’. The capitalized sentence was present at the bottom of the screen throughout the transfer test. By contrast, the non-instructed group received the same task but the capitalized sentence was omitted throughout. In each trial, either the beer stimulus (a picture showing two glass jugs of beer being clashed together – identical to experiment 2) or a chocolate stimulus (a close up of Cadbury chocolate chunks), or no stimulus, was randomly selected for presentation for 1-s prior to the prompt ‘← or →’, across 48 trials. The dependent measure was the percentage choice of the beer (R1) vs. chocolate response (R2). It was expected that in the instructed group, the beer and chocolate stimuli would show less control over responding for the corresponding outcome compared to the non-instructed group (Fig. 5) demonstrating the role of propositional hierarchical instrumental knowledge in driving the PIT effect.

#### Hierarchical beliefs

**Fig. 4:**
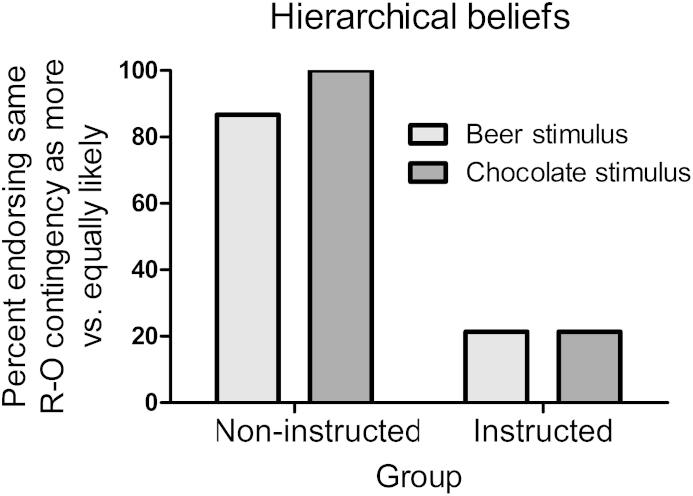
Percent participants that endorsed the belief that the corresponding response-outcome relationship was more likely vs. equally likely to be rewarded in the beer and chocolate stimulus. The uninstructed group believed that each stimulus signalled a greater likelihood of the corresponding response being reinforced, whereas only a small percentage of the instructed group, who were told that this was not the case, retained this belief.

Participants' beliefs about the hierarchical relationships operating in the PIT task were assessed immediately afterwards. They were shown the beer and chocolate stimuli in random order on the screen, and asked ‘When this picture was on the screen, did you think that the *same reward key* [‘beer key’/‘chocolate key’] was (1) more likely or (2) equally likely to be rewarded compared to the *different reward key* [‘beer key’/‘chocolate key’]’. The dependent measure was the percentage of participants who endorsed the belief that the same reward key was more likely to be rewarded, rather than equally likely to be rewarded, in each stimulus. Instructions were expected to decrease the proportion of participants endorsing the hierarchical beliefs (Fig. 4).

### Results

As shown in Fig. 4, nearly 100% of the non-instructed group believed that during the PIT test each stimulus signalled that the response that produced the corresponding outcome was *more likely* to be rewarded than the response that produced the different outcome. By contrast, significantly fewer participants in the instructed group endorsed these beliefs (chi square *p*s < .001), instead they predominately endorsed the ‘*equally likely*’ statement in each stimulus. Thus, the instructions were effective in degrading hierarchical beliefs.

**Fig. 5:**
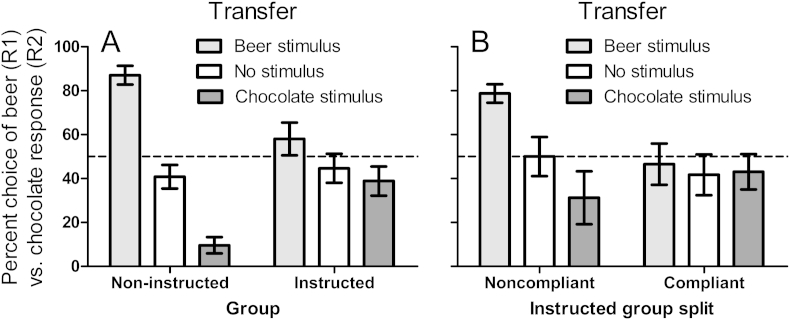
(A) Mean percent choice between a beer response (R1) vs. chocolate response (R2) (±sem) in a beer, chocolate and no stimulus condition, in the transfer test, for the instructed and non-instructed group. The PIT effect was abolished by instructions stating that stimuli did not signal which R-O contingency was more effective. (B) The same PIT data but with the instructed group split into a non-compliant sub-group who retained hierarchical beliefs despite instructions to the contrary, and a compliant sub-group who abandoned their hierarchical beliefs in accordance with the instructions. Both figures A and B show that the PIT effect depends upon propositional hierarchical expectancies.

As shown in Fig. 5A, the instructions also abolished the PIT effect. While the non-instructed group showed a standard PIT effect (where the beer and chocolate stimuli primed the beer and chocolate response, respectively), this PIT effect was absent in the instructed group. ANOVA on Fig. 5A yielded a significant interaction between stimulus and group, *F*(2,54) = 15.21, *p* < .001, where the effect of stimulus was significant in the non-instructed group, *F*(2,28) = 63.26, *p* < .001, and was marked but non-significant in the instructed group, *F*(2,26) = 2.97, *p* < .07.

On closer examination of the instructed group, it was found that two participants endorsed the hierarchical ‘more likely’ statement in the beer stimulus, two endorsed this belief in the chocolate stimulus, and one endorsed this belief in both stimuli. Thus, 5 out of 14 (36%) of instructed participants reported some hierarchical beliefs contrary to instructions. These participants were labelled as a non-compliant sub-group, and as Fig. 5B indicates, they showed a PIT effect whereas the compliant sub-group (who endorsed the ‘equally likely’ statement in both stimuli) did not. ANOVA on Fig. 5B yielded a significant interaction between stimulus and instructed sub-group, *F*(2,24) = 4.28, *p* = .03, where the main effect of stimulus was significant in the non-compliant sub-group, *F*(2,8) = 7.97, *p* = .02, and non-significant in the compliant sub-group, *F* < 1.

### Discussion

Experiment 3 showed that instructions which stated that no hierarchical relations existed in the PIT test abolished both participants' propositional hierarchical beliefs and the PIT effect. Furthermore, only those instructed participants who completely abandoned their propositional hierarchical beliefs showed abolition of the PIT effect, whereas those who retained some hierarchical beliefs showed a PIT effect, confirming the tight correspondence between hierarchical beliefs and cue priming of action selection.

## General discussion

The current set of studies found that the ability of drug cues to transfer control over a separately trained drug-seeking response was not abolished by Pavlovian extinction where the stimulus was presented without the drug (S-no O), but was abolished by discriminative extinction training where the stimulus signalled that the response-drug contingency would be nonreinforced (S:R-no O). The reduced effectiveness of Pavlovian compared to discriminative extinction training in abolishing the PIT effect confirms previous studies with natural rewards in humans and animals (Delamater, 1996; Gámez & Rosas, 2005; Rescorla, 1992a; Rosas et al., 2010). These findings also mirror the observation that Pavlovian stimuli produce smaller PIT effects than discriminative stimuli (Rescorla, 1994a; Troisi, 2006 see also; Holman & Mackintosh, 1981), confirming that PIT is driven by the discriminative or hierarchical function of cues. Finally, the consistent finding of a dissociable effect of Pavlovian and discriminative extinction training on PIT in animal and human non-drug designs suggests that this same dissociation found between Experiment 1 and 2 was not merely due to the shift from smokers to drinkers, but due to the two extinction methods employed.

Additionally, experiment 3 found that participants who abandoned their hierarchical beliefs following instructions that stimuli did *not* signal which R-O contingency was more likely to be rewarded, showed no PIT effect. By contrast, participants who possessed these hierarchical beliefs (the non-instructed group and non-compliant instructed sub-group) showed a PIT effect. This dependency of the PIT effect on hierarchical beliefs supports the claim that transfer of stimulus control over instrumental performance can be propositional in nature (Heyes & Dickinson, 1990; Mitchell, De Houwer, & Lovibond, 2009), where the S retrieves a verbalizable gestalt R-O expectancy, which drives selection of the response (Colwill & Delamater, 1995; Colwill & Rescorla, 1990b; Rescorla, 1991). These findings support hierarchical, cognitive, strategic, propositional, behavioural-economic accounts of drug cue-reactivity over accounts which claim automaticity, implicit associations, stimulus-response habits, or Pavlovian conditioned responses to be the underlying mechanism (Troisi, 2013b).

The claim that external discriminative stimuli prime responding by retrieving a gestalt response-outcome expectancy is supported by a diverse variety of findings. These finding include: Affordance, where stimuli immediately provoke responses relevant to the outcome (Şahin, Çakmak, Doğar, Uğur, & Üçoluk, 2007); The selection between response options on the basis of net pay-off, which reflects the expected reward minus response costs (Kennerley & Walton, 2011); Response-compatibility effects where stimuli elicit responses more quickly if the stimulus is compatible with the outcome expected from the response with respect to spatial position (Hommel, 1993; Kunde, 2001; Lu & Proctor, 1995), affective code (Eder, Müsseler, & Hommel, 2012), perceptual identity or semantic meaning (Koch & Kunde, 2002); The faster acquisition of instrumental discriminations if the stimulus is compatible with the outcome expected from the response with respect to spatial position (Overmier, Bull, & Trapold, 1971; Trapold, 1970; Urcuioli, 2005; see also; Rescorla & Cunningham, 1979) or perceptual identity (Dwyer, Dunn, Rhodes, & Killcross, 2010; de Wit, Ridderinkhof, Fletcher, & Dickinson, 2012). The implication of these studies is that drug stimuli will most readily retrieve a representation of the response-drug contingency by virtue of possessing spatial, affective, perceptual or semantic elements in common with the drug outcome, and such ‘compatible’ stimuli will be most hazardous in producing relapse. One applied illustration of this analysis comes from the introduction of standardized (plain) packaging for cigarettes. Speculatively, this policy may promote abstinence (Wakefield, Hayes, Durkin, & Borland, 2013) by degrading the common affective or perceptual elements between the packaging (stimulus) and the outcome (smoking reward), such that the packaging does not invite (afford) the act of smoking.

The hierarchical account of stimulus control can also be integrated with ideas about how the outcome's current value guides action selection (Balleine & Ostlund, 2007; de Wit & Dickinson, 2009). It has been found that in free-operant conditions response choice is sensitive to the value of the outcomes on offer, demonstrating goal-directed control over action selection. Paradoxically, however, when a single stimulus is presented which signals that one R-O contingency is stronger, as in the PIT test, this stimulus tends to enhance the propensity to make the corresponding response with a magnitude which is itself not weighted by the current value of the outcome. In other words, stimuli may prime action despite the expected outcome having little or no value (Colwill & Rescorla, 1990a; Corbit, Janak, & Balleine, 2007; Holland, 2004; Rescorla, 1994b). Such cue-priming of action is pathological in the sense of not being constrained by the desires of the individual. The relevance of this analysis for addiction is clear; cue-evoked drug-seeking in the PIT test is autonomous of devaluation of the drug produced by satiety, health warning (Hogarth & Chase, 2011) and pharmacotherapy (Ferguson & Shiffman, 2009; Hitsman et al., 2013; Hogarth, 2012), and cue-evoked drug consumption is similarly autonomous of satiety (Hogarth et al., 2010). The implication is that the hierarchical priming function of drug stimuli, despite apparently being propositional in nature, nevertheless represents a pathological form of control over drug-seeking which may promote binging despite satiety, and relapse despite desire to quit, knowledge of drug related harms, or pharmacological devaluation of the drug.

The therapeutic potential of discriminative extinction training has been encapsulated in a quote from Rescorla (1991, page 21): ‘one might assume that an instrumental discriminative stimulus gains control over responding to the degree that the R-O association undergoes an increase in its presence. Conversely, that stimulus might lose control over responding whenever the R-O association undergoes a decrease in its presence’. Discriminative extinction training in Experiment 2 proved this second claim; the question is whether such training could have therapeutic effects beyond the paradigm. Some insight into this possibility may be drawn from three broadly related retraining approaches: Attentional retraining (Attwood, O'Sullivan, Leonards, Mackintosh, & Munafo, 2008; Field et al., 2007; Schoenmakers et al., 2010), inhibitory control training (Houben, Nederkoorn, Wiers, & Jansen, 2011; Jones & Field, 2013; Veling, Aarts, & Stroebe, 2013; Verbruggen & Logan, 2008) and avoidance training (Eberl et al., 2013). Whereas attentional retraining has failed to deliver therapeutic effects (anticipated by some preclinical models: Hogarth, Dickinson, & Duka, 2009; Hogarth, Dickinson, Janowski, Nikitina, & Duka, 2008), inhibitory control training currently has equivocal evidence of clinical efficacy, and avoidance training has some positive evidence that awaits substantiation. Avoidance training has perhaps the greatest similarity to discriminative extinction training used here, insofar as instructions require that in the drug stimulus participants should produce a new avoidance response which causes the drug cue to shrink/recede (S:R-less O). Generally, it is fair to say that these retraining methods are yet to produce substantial therapeutic effects.

For discriminative extinction learning to yield therapeutic effects, at least three technical challenges must be overcome. The first challenge is to enhance the generalisation of extinction learning across contexts. Extinction learning is typically found to be context specific (Rosas, Todd, & Bouton, 2013). If contingencies are acquired in context A, and extinction learning occurs in context B, extinction learning typically does not transfer substantially to context A. Because discriminative control of responding can come under contextual governance (Haddon, George, & Killcross, 2008), it is likely that discriminative extinction training would similarly come under contextual governance. In short, there is no reason to suppose that discriminative extinction learning possesses special facility to transfer across contexts.

The second challenge is to enhance the generalisation of extinction learning to other equivalent stimuli and responses. Experiment 2 suggested that participants generalised the extinction rule S1:R1-no O, *proactively* to a subsequently acquired equivalent response, inferring S1:R3-no O. A key question is whether extinction training with a subsequently acquired response, S1:R3-no O, would generalise *retroactively* to a previously acquired response, to produce the inference S1:R1-no O. Similarly, it is not known whether discriminative extinction learning would generalise (proactively or retroactively) to novel equivalent drug stimuli. The results from animal studies using the ABA renewal paradigm speak to these issues. Such studies have shown that Pavlovian extinction of the renewal context (A) does not attenuate the renewal affect (Bouton, Todd, Vurbic, & Winterbauer, 2011, experiment 4), consistent with no effect of Pavlovian extinction training on PIT (experiment 1). By contrast, ‘discriminative extinction’ training where the renewal context served as the stimulus signalling that the response would not be rewarded (S:R1-no O), did attenuate renewal of that response in the renewal context (Todd, Vurbic, & Bouton, in press), consistent with the impact of discriminative extinction training on PIT (experiment 2). Crucially for the retroactive generalisation issue, however, discriminative extinction learning in context A did not generalise retroactively to attenuate renewal of a previously established response trained in that context (Todd, 2013, experiment 4; Troisi, LeMay, & Järbe, 2010). If discriminative extinction training does not generalise retroactively to established responses, how could such training impact on established drug-seeking?

Additional components might be added to boost generalisation of extinction learning. For example, Millan, Milligan-Saville, & McNally (2013) found that renewal of nose poking for beer in the ABA paradigm was better attenuated by segmented blocks of discriminative extinction training than a continuous block of the same overall length. Similarly, conducting extinction learning in multiple contexts enhanced generalisation of the extinction effect (Glautier & Elgueta, 2009; Gunther, Denniston, & Miller, 1998). However, it is not known whether degrading context specificity in this way would enhance the generalisation of extinction learning to equivalent stimuli and responses, either retroactively or proactively. The most compelling future direction for this research, therefore, would be to determine whether discriminative extinction training conducted with multiple stimuli, responses and contexts, would enhance generalisation to novel experimental configurations. Only once this has been achieved, might one anticipate therapeutic efficacy in the natural environment.

Several limitations of the current findings further challenge their clinical application. First, each experiment recruited a small heterogeneous sample of sub-clinical drug users. One may question whether the propositional form of drug stimulus control demonstrated in the current sub-clinical sample also operates for clinical populations who are more severally dependent or have accrued neuro-cognitive or psychiatric damage (Goldstein & Volkow, 2011). Consistent with this claim, chronic drug exposure has been shown to attenuate specific PIT effects in rats (Shiflett, 2012; although not in human alcoholics Garbusow et al., in press), promoting a transition to more general form of stimulus control wherein stimuli modify instrumental performance by eliciting a general motivational state rather than by retrieving specific response-outcome expectancies (Corbit et al., 2007; Hogarth et al., 2013; Nadler, Delgado, & Delamater, 2011). It remains unknown whether discriminative extinction training would modify this form of general PIT. Another crucial feature of clinical compared to non-clinical samples is their greater motivation to quit, which has been shown to improve the effectiveness of pharmacotherapy (Perkins et al., 2010) and change the neural response to drug cues (Wilson, Sayette, & Fiez, 2012). It remains unknown how motivation to quit would interact with discriminative extinction methods, particularly generalisation of extinction across contexts, but this is a potentially valuable line of inquiry. Conversely, clinical drug users, especially during relapse, have been characterised as being subject to hot or visceral cognition (i.e. intense, seemingly uncontrollable cravings) which drive drug-seeking (Loewenstein, 1996). By contrast, participants in the current studies responded for the drug on average in less than 50% of trials indicating relative indifference towards the drug over the natural reinforcer. It remains unknown to what extent discriminative extinction of cues would counter drug-motivation arising from such interoceptive or craving states. Indeed, there is evidence that extinction learning is not only specific to the external context (as outlined above), but also specific to internal state in which learning takes place (Troisi, 2013a). Thus, extinction learning may be lost following a transition to a ‘hot’ internal state. In sum, although the application of the current findings to clinical therapeutics raises many unanswered questions, the demonstration of a more effective drug cue extinction method, and its propositional basis, may nevertheless invigorate interest in this field of study.

To conclude, the studies reported here found that transfer of drug stimulus control over a separately acquired drug-seeking response was not abolished by Pavlovian extinction (S-no O), but was abolished by discriminative extinction training (S:R-no O), and by propositional hierarchical instructions stating that the drug stimulus did not signal that drug-seeking was more likely to be rewarded. These data suggest that therapies which degrade the ability of drug cues to retrieve a response-drug expectancy should be superior to Pavlovian cue exposure therapies. However, a better understanding of how to enhance the generalisation of discriminative extinction learning to other stimuli, responses and internal/external contexts is required to realise the therapeutic potential of this technique.

## Figures and Tables

**Table 1:** Design of experiment 1.

Concurrent training	Pavlovian acquisition	Pavlovian extinction	Transfer test
R1-O1 R2-O2	S1-O1 S2-O1 S3-O2 S4-O2	S1-O1 S2-no O1 S3-O2 S4-no O2	S1:R1/R2 S2:R1/R2 S3:R1/R2 S4:R1/R2

R1/R2 = keyboard response; S = conditioned stimuli; O1 = Tobacco points; O2=Chocolate points.

**Table 2:** Design of experiment 2.

Concurrent training 1	Discriminative extinction training	Concurrent training 2	Transfer test
**Extinguished group**			
R1-O1	S1: R1-no O1, R2-O2	R3-O1	S1:R3/R4
R2-O2	S2: R1-O1, R2-O2	R4-O2	S2:R3/R4
**Non-extinguished group**			
R1-O1	S1: R1-O1, R2-O2	R3-O1	S1:R3/R4
R2-O2	S2: R1-O1, R2-O2	R4-O2	S2:R3/R4

R1/R2 = keyboard responses (up or down arrow key); S1 = beer picture; S2 = no stimulus; R3/R4 = keyboard responses (A or D key); O1 = Beer points, O2 = Chocolate points.

**Table 3:** Design of experiment 3.

Concurrent training	Instructions	Transfer test
**Instructed group**		
R1-O1 R2-O2	“Pictures do not indicate which key is more likely to be rewarded!”	S1:R1/R2 S2:R1/R2 S3:R1/R2
**Non-instructed group**		
R1-O1 R2-O2		S1:R1/R2 S2:R1/R2 S3:R1/R2

R1/R2 = keyboard responses; S1 = beer picture; S2 = no stimulus; S3 = chocolate picture; O1 = Beer points, O2 = Chocolate points.

## References

[bib1] Attwood A.S., O'Sullivan H., Leonards U., Mackintosh B., Munafo M.R. (2008). Attentional bias training and cue reactivity in cigarette smokers. Addiction.

[bib2] Babor T.F., Higgins-Biddle J.C., Saunders J.B., Monteiro M.G. (2001). AUDIT: The alcohol use disorders identification test guidelines for use in primary care.

[bib3] Balleine B.W., Ostlund S.B., Balleine B.W., Doya K., Doherty J.O., Sakagami M. (2007). Still at the choice-point: action selection and initiation in instrumental conditioning. Reward and decision making in corticobasal ganglia networks.

[bib4] Bouton M.E., Todd T.P., Vurbic D., Winterbauer N.E. (2011). Renewal after the extinction of free operant behavior. Learning & Behavior.

[bib5] Bradfield L.A., Balleine B.W. (2013). Hierarchical and binary associations compete for behavioral control during instrumental biconditional discrimination. Journal of Experimental Psychology: Animal Behavior Processes.

[bib6] Cisek P. (2007). Cortical mechanisms of action selection: the affordance competition hypothesis. Philosophical Transactions of the Royal Society B: Biological Sciences.

[bib7] Collins B.N., Brandon T.H. (2002). Effects of extinction context and retrieval cues on alcohol cue reactivity among nonalcoholic drinkers. Journal of Consulting and Clinical Psychology.

[bib8] Colwill R.M., Douglas L.M. (1994). Associative representations of instrumental contingencies.

[bib9] Colwill R.M., Delamater B.A. (1995). An associative analysis of instrumental biconditional discrimination learning. Animal Learning & Behavior.

[bib10] Colwill R.M., Rescorla R.A. (1990). Effects of reinforcer devaluation on discriminative control of instrumental behavior. Journal of Experimental Psychology: Animal Behavior Processes.

[bib11] Colwill R.M., Rescorla R.A. (1990). Evidence for the hierarchical structure of instrumental learning. Animal Learning & Behavior.

[bib12] Conklin C.A., Tiffany S.T. (2002). Applying extinction research and theory to cue-exposure addiction treatments. Addiction.

[bib13] Corbit L.H., Janak P.H., Balleine B.W. (2007). General and outcome-specific forms of Pavlovian-instrumental transfer: the effect of shifts in motivational state and inactivation of the ventral tegmental area. European Journal of Neuroscience.

[bib14] Delamater A.R. (1995). Outcome-selective effects of intertrial reinforcement in Pavlovian appetitive conditioning with rats. Animal Learning & Behavior.

[bib15] Delamater A.R. (1996). Effects of several extinction treatments upon the integrity of Pavlovian stimulus-outcome associations. Learning & Behavior.

[bib16] Dwyer D.M., Dunn M.J., Rhodes S.E.V., Killcross A.S. (2010). Lesions of the prelimbic prefrontal cortex prevent response conflict produced by action-outcome associations. The Quarterly Journal of Experimental Psychology.

[bib17] Eberl C., Wiers R.W., Pawelczack S., Rinck M., Becker E.S., Lindenmeyer J. (2013). Approach bias modification in alcohol dependence: do clinical effects replicate and for whom does it work best?. Developmental Cognitive Neuroscience.

[bib18] Eder A., Müsseler J., Hommel B. (2012). The structure of affective action representations: temporal binding of affective response codes. Psychological Research.

[bib19] Ferguson S.G., Shiffman S. (2009). The relevance and treatment of cue-induced cravings in tobacco dependence. Journal of Substance Abuse Treatment.

[bib20] Field M., Duka T., Eastwood B., Child R., Santarcangelo M., Gayton M. (2007). Experimental manipulation of attentional biases in heavy drinkers: do the effects generalise?. Psychopharmacology.

[bib21] Gámez A.M., Rosas J.M. (2005). Transfer of stimulus control across instrumental responses is attenuated by extinction in human instrumental conditioning. International Journal of Psychology & Psychological Therapy.

[bib22] Garbusow, M., Schad, D. J., Sommer, C., Jünger, E., Sebold, M., Friedel, E., et al. Pavlovian-to-instrumental-transfer in alcohol dependence – a pilot study. *Neuropsychobiology* (in press). 10.1159/000363507 25359491

[bib23] Gass J.T., Chandler L.J. (2013). The plasticity of extinction: contribution of the prefrontal cortex in treating addiction though inhibitory learning. Frontiers in Psychiatry.

[bib24] Glautier S., Elgueta T. (2009). Multiple cue extinction effects on recovery of responding in causal judgments. International Journal of Comparative Psychology.

[bib25] Goldstein R.Z., Volkow N.D. (2011). Dysfunction of the prefrontal cortex in addiction: neuroimaging findings and clinical implications. Nature Reviews Neuroscience.

[bib26] Gunther L.M., Denniston J.C., Miller R.R. (1998). Conducting exposure treatment in multiple contexts can prevent relapse. Behaviour Research and Therapy.

[bib27] Haddon J.E., George D.N., Killcross S. (2008). Contextual control of biconditional task performance: evidence for cue and response competition in rats. Quarterly Journal of Experimental Psychology.

[bib28] Heyes C., Dickinson A. (1990). The intentionality of animal action. Mind and Language.

[bib29] Hitsman B., Hogarth L., Tseng L.-J., Teige J.C., Shadel W.G., DiBenedetti D.B. (2013). Dissociable effect of acute varenicline on tonic versus cue-provoked craving in non-treatment-motivated heavy smokers. Drug and Alcohol Dependence.

[bib30] Hogarth L. (2012). Goal-directed and transfer-cue-elicited drug-seeking are dissociated by pharmacotherapy: evidence for independent additive controllers. Journal of Experimental Psychology: Animal Behavior Processes.

[bib31] Hogarth L., Balleine B.W., Corbit L.H., Killcross S. (2013). Associative learning mechanisms underpinning the transition from recreational drug use to addiction. Annals of the New York Academy of Sciences.

[bib32] Hogarth L., Chase H.W. (2011). Parallel goal-directed and habitual control of human drug-seeking: implications for dependence vulnerability. Journal of Experimental Psychology: Animal Behavior Processes.

[bib33] Hogarth L., Chase H.W. (2012). Evaluating psychological markers for human nicotine dependence: tobacco choice, extinction, and Pavlovian-to-instrumental transfer. Experimental and Clinical Psychopharmacology.

[bib34] Hogarth L., Dickinson A., Duka T. (2009). Detection versus sustained attention to drug cues have dissociable roles in mediating drug seeking behaviour. Experimental and Clinical Psychopharmacology.

[bib35] Hogarth L., Dickinson A., Duka T. (2010). The associative basis of cue elicited drug taking in humans. Psychopharmacology.

[bib36] Hogarth L., Dickinson A., Janowski M., Nikitina A., Duka T. (2008). The role of attentional bias in mediating human drug seeking behaviour. Psychopharmacology.

[bib37] Hogarth L., Dickinson A., Wright A., Kouvaraki M., Duka T. (2007). The role of drug expectancy in the control of human drug seeking. Journal of Experimental Psychology: Animal Behavior Processes.

[bib38] Holland P.C. (2004). Relations between Pavlovian-instrumental transfer and reinforcer devaluation. Journal of Experimental Psychology: Animal Behavior Processes.

[bib39] Holman J.G., Mackintosh N.J. (1981). The control of appetitive instrumental responding does not depend on classical conditioning to the discriminative stimulus. Quarterly Journal of Experimental Psychology Section B.

[bib40] Holmes N.M., Marchand A.R., Coutureau E. (2010). Pavlovian to instrumental transfer: a neurobehavioural perspective. Neuroscience & Biobehavioral Reviews.

[bib41] Hommel B. (1993). Inverting the Simon effect by intention. Psychological Research.

[bib42] Houben K., Nederkoorn C., Wiers R.W., Jansen A. (2011). Resisting temptation: decreasing alcohol-related affect and drinking behavior by training response inhibition. Drug and Alcohol Dependence.

[bib43] Jones A., Field M. (2013). The effects of cue-specific inhibition training on alcohol consumption in heavy social drinkers. Experimental and Clinical Psychopharmacology.

[bib44] Kennerley S.W., Walton M.E. (2011). Decision making and reward in frontal cortex: complementary evidence from neurophysiological and neuropsychological studies. Behavioral Neuroscience.

[bib45] Koch I., Kunde W. (2002). Verbal response-effect compatibility. Memory & Cognition.

[bib46] Kunde W. (2001). Response-effect compatibility in manual choice reaction tasks. Journal of Experimental Psychology: Human Perception and Performance.

[bib47] Loewenstein G. (1996). Out of control: visceral influences on behavior. Organizational Behavior and Human Decision Processes.

[bib48] Lu C.H., Proctor R.W. (1995). The influence of irrelevant location information on performance: a review of the Simon and spatial Stroop effects. Psychonomic Bulletin & Review.

[bib49] Martinovic J., Jones A., Christiansen P., Rose A.K., Hogarth L., Field M. (2014). Electrophysiological responses to alcohol cues are not associated with Pavlovian-to-instrumental transfer in social drinkers. Plos One.

[bib50] Millan E.Z., Milligan-Saville J., McNally G.P. (2013). Memory retrieval, extinction, and reinstatement of alcohol seeking. Neurobiology of Learning and Memory.

[bib51] Mitchell C.J., De Houwer J., Lovibond P.F. (2009). The propositional nature of human associative learning. Behavioral and Brain Sciences.

[bib52] Nadler N., Delgado M.R., Delamater A.R. (2011). Pavlovian to instrumental transfer of control in a human learning task. Emotion.

[bib53] Overmier J.B., Bull J.A., Trapold M.A. (1971). Discriminative cue properties of different fears and their role in response selection in dogs. Journal of Comparative and Physiological Psychology.

[bib54] Perkins K.A., Lerman C., Fonte C.A., Mercincavage M., Stitzer M.L., Chengappa K.N.R. (2010). Cross-validation of a new procedure for early screening of smoking cessation medications in humans. Clinical Pharmacology and Therapeutics.

[bib55] Price K.L., Saladin M.E., Baker N.L., Tolliver B.K., DeSantis S.M., McRae-Clark A.L. (2010). Extinction of drug cue reactivity in methamphetamine-dependent individuals. Behaviour Research and Therapy.

[bib56] Rescorla R.A. (1991). Associative relations in instrumental learning – the 18 Bartlett memorial lecture. Quarterly Journal of Experimental Psychology Section B – Comparative and Physiological Psychology.

[bib57] Rescorla R.A. (1992). Associations between an instrumental discriminative stimulus and multiple outcomes. Journal of Experimental Psychology: Animal Behavior Processes.

[bib58] Rescorla R.A. (1992). Response-outcome versus outcome-response associations in instrumental learning. Animal Learning & Behavior.

[bib59] Rescorla R.A. (1994). Control of instrumental performance by Pavlovian and instrumental stimuli. Journal of Experimental Psychology: Animal Behavior Processes.

[bib60] Rescorla R.A. (1994). Transfer of instrumental control mediated by a devalued outcome. Animal Learning & Behavior.

[bib61] Rescorla R.A., Cunningham C.L. (1979). Spatial contiguity facilitates Pavlovian second-order conditioning. Journal of Experimental Psychology: Animal Behavior Processes.

[bib62] Rosas J.M., Paredes-Olay M.C., García-Gutiérrez A., Espinosa J.J., Abad M.J.F. (2010). Outcome-specific transfer between predictive and instrumental learning is unaffected by extinction but reversed by counterconditioning in human participants. Learning and Motivation.

[bib63] Rosas J.M., Todd T.P., Bouton M.E. (2013). Context change and associative learning. Wiley Interdisciplinary Reviews: Cognitive Science.

[bib64] Şahin E., Çakmak M., Doğar M.R., Uğur E., Üçoluk G. (2007). To afford or not to afford: a new formalization of affordances toward affordance-based robot control. Adaptive Behavior.

[bib65] Sayette M.A., Tiffany S.T. (2013). Peak provoked craving: an alternative to smoking cue-reactivity. Addiction.

[bib66] Schoenmakers T.M., de Bruin M., Lux I.F.M., Goertz A.G., Van Kerkhof D.H.A.T., Wiers R.W. (2010). Clinical effectiveness of attentional bias modification training in abstinent alcoholic patients. Drug and Alcohol Dependence.

[bib67] Shiffman S. (2009). Responses to smoking cues are relevant to smoking and relapse. Addiction.

[bib68] Shiflett M. (2012). The effects of amphetamine exposure on outcome-selective Pavlovian-instrumental transfer in rats. Psychopharmacology.

[bib69] Thewissen R., Snijders S.J.B.D., Havermans R.C., van den Hout M., Jansen A. (2006). Renewal of cue-elicited urge to smoke: Implications for cue exposure treatment. Behaviour Research and Therapy.

[bib70] Todd T.P. (2013). Mechanisms of renewal after the extinction of instrumental behavior. Journal of Experimental Psychology: Animal Behavior Processes.

[bib71] Todd T.P., Vurbic D., Bouton M.E. (2014). Behavioral and neurobiological mechanisms of extinction in Pavlovian and instrumental learning. Neurobiology of Learning and Memory.

[bib72] Trapold M.A. (1970). Are expectancies based upon different positive reinforcing events discriminably different?. Learning and Motivation.

[bib73] Troisi J.R.I. (2006). Pavlovian-instrumental transfer of the discriminative stimulus effects of nicotine and ethanol in rats. Psychological Record.

[bib74] Troisi J.R.I. (2013). Acquisition, extinction, recovery, and reversal of different response sequences under conditional control by nicotine in rats. The Journal of General Psychology.

[bib75] Troisi J.R.I. (2013). Perhaps more consideration of Pavlovian operant interactions may improve the clinical efficacy of behaviorally based drug treatment programs. Psychological Record.

[bib76] Troisi J.R.I., LeMay B.J., Järbe T.U.C. (2010). Transfer of the discriminative stimulus effects of Δ9-THC and nicotine from one operant response to another in rats. Psychopharmacology.

[bib77] Urcuioli P.J. (2005). Behavioral and associative effects of differential outcomes in discrimination learning. Animal Learning & Behavior.

[bib78] Veling H., Aarts H., Stroebe W. (2013). Using stop signals to reduce impulsive choices for palatable unhealthy foods. British Journal of Health Psychology.

[bib79] Verbruggen F., Logan G.D. (2008). Automatic and controlled response inhibition: associative learning in the go/no-go and stop-signal paradigms. Journal of Experimental Psychology: General.

[bib80] Wakefield M.A., Hayes L., Durkin S., Borland R. (2013). Introduction effects of the Australian plain packaging policy on adult smokers: a cross-sectional study. BMJ Open.

[bib81] Wilson S.J., Sayette M.A., Fiez J.A. (2012). Quitting-unmotivated and quitting-motivated cigarette smokers exhibit different patterns of cue-elicited brain activation when anticipating an opportunity to smoke. Journal of Abnormal Psychology.

[bib82] de Wit S., Dickinson A. (2009). Associative theories of goal-directed behaviour: a case for animal–human translational models. Psychological Research.

[bib83] de Wit S., Ridderinkhof K.R., Fletcher P., Dickinson A. (2012). Resolution of outcome-induced response conflict by humans after extended training. Psychological Research.

[bib84] Xue Y.X., Luo Y.X., Wu P., Shi H.S., Xue L.F., Chen C. (2012). A memory retrieval-extinction procedure to prevent drug craving and relapse. Science.

